# Nurses’ Roles in mHealth App Development: Scoping Review

**DOI:** 10.2196/46058

**Published:** 2023-10-17

**Authors:** Caitlin J Bakker, Tami H Wyatt, Melissa CS Breth, Grace Gao, Lisa M Janeway, Mikyoung A Lee, Christie L Martin, Victoria L Tiase

**Affiliations:** 1 Dr John Archer Library University of Regina Regina, SK Canada; 2 College of Nursing University of Tennessee Knoxville Knoxville, TN United States; 3 Clinical Quality Informatics, The Joint Commission Oakbrook Terrace, IL United States; 4 School of Nursing St. Catherine University St Paul, MN United States; 5 National Veterans Affairs Quality Scholars Program Joseph Maxwell Cleland Atlanta Veterans Affairs Medical Center Atlanta, GA United States; 6 Northwestern Medicine Chicago, IL United States; 7 Oak Point University Oak Brook, IL United States; 8 College of Nursing Texas Woman's University Dallas, TX United States; 9 School of Nursing University of Minnesota Minneapolis, MN United States; 10 Department of Biomedical Informatics University of Utah Salt Lake City, UT United States

**Keywords:** mobile health, mHealth, mobile app, product development, software design, scoping, search strategy, nursing, health app, mobile app, nurse, nursing, allied health, development, design, software, scoping literature review, scoping review, app, sensor, wearable, software development, mobile phone

## Abstract

**Background:**

Although mobile health (mHealth) apps for both health consumers and health care providers are increasingly common, their implementation is frequently unsuccessful when there is a misalignment between the needs of the user and the app’s functionality. Nurses are well positioned to help address this challenge. However, nurses’ engagement in mHealth app development remains unclear.

**Objective:**

This scoping review aims to determine the extent of the evidence of the role of nurses in app development, delineate developmental phases in which nurses are involved, and to characterize the type of mHealth apps nurses are involved in developing.

**Methods:**

We conducted a scoping review following the 6-stage methodology. We searched 14 databases to identify publications on the role of nurses in mHealth app development and hand searched the reference lists of relevant publications. Two independent researchers performed all screening and data extraction, and a third reviewer resolved any discrepancies. Data were synthesized and grouped by the Software Development Life Cycle phase, and the app functionality was described using the IMS Institute for Healthcare Informatics functionality scoring system.

**Results:**

The screening process resulted in 157 publications being included in our analysis. Nurses were involved in mHealth app development across all stages of the Software Development Life Cycle but most frequently participated in design and prototyping, requirements gathering, and testing. Nurses most often played the role of evaluators, followed by subject matter experts. Nurses infrequently participated in software development or planning, and participation as patient advocates, research experts, or nurse informaticists was rare.

**Conclusions:**

Although nurses were represented throughout the preimplementation development process, nurses’ involvement was concentrated in specific phases and roles.

## Introduction

### Background

More than 350,000 mobile health (mHealth) apps are available in major app stores worldwide, including medical, health care, and fitness apps [1]. Although there is no standardized definition of mHealth, it broadly refers to health care supported by mobile and wireless devices to deliver, educate, and exchange health care information and monitor and promote health conditions or behaviors [2-4]. Many of these apps are focused on condition management, such as mental and behavioral health disorders, diabetes, and heart and circulatory system conditions [1]. One survey of American adults who had used an mHealth app found that approximately 70% of them had used it to keep track of their health, 39% to obtain health information, and 25% to share health information with their providers [5]. A Pew Research Center study reported that 62% of smartphone users used their devices to gather health-related information [6], and 90% of physicians used smartphones at work to access electronic health records, communicate with their team, reference information, or manage their schedule [7]. Similar to physicians, many nurses use apps for professional purposes [8].

Despite the ubiquity of mHealth apps for health care consumers and providers, health information technology implementation is frequently unsuccessful [9]. Previous research has found that apps may not align with end-user behavior and organizational needs, essentially showing a mismatch between how the app is designed to function and how the intended end user expects it to work [10-12].

Nurses are uniquely positioned to help address the challenge of this mismatch. The American Nurses Association Code of Ethics for Nurses includes multiple provisions emphasizing the centrality of the patient in health care, stating that “[t]he nurse’s primary commitment is to the patient” and that “[t]he nurse promotes, advocates for, and protects the rights, health, and safety of the patient” [13]. Integrating nurses as product development team members brings this patient-centered perspective to the development process. It has the potential to create apps that contain validated, current evidence-based health-related content that is meaningful to end users. Nurse informaticists, in particular, can ensure that the usability and features of the app are relevant for all end users and are incorporated into clinical workflows, leveraging interoperability standards [14].

Despite this potential, the extent to which nurses are involved in all aspects of the development process remains unclear. A thorough understanding of the role of nurses in mHealth app development is critical for several reasons. The first and most essential is that nursing professionals represent the largest segment of the health care workforce, spend the most time with patients, and coordinate all aspects of patient care [15]. Previous research has found that physicians in the intensive care unit spend 15% to 18% of their time with patients, whereas nurses in the same study spent 33% of their time in patient rooms and an additional 11% to 12% of their time directly outside patient rooms [16]. A longitudinal study of hospital nurses found that nurses spend 37% of their time with patients, and that direct care, indirect care, medication management, and communicating with other health professionals consumed >76% of the nurses’ time [17]. Therefore, nurses have a holistic view of health care processes and are keenly aware of what problems need to be solved.

### Objectives

To better understand nurses’ role in mHealth app development, this scoping review aims to determine the extent of the evidence regarding the role of nurses in app development and describe the apps nurses are involved in developing by answering the following research questions: (1) what role or roles do nurses perform in mHealth app development? (2) in what phases are nurses involved in mHealth app development? and (3) what type of apps are nurses involved in developing?

## Methods

### Overview

A scoping review was selected as the appropriate methodology, as the objectives of the project were to determine the extent of the evidence and to identify gaps in the existing literature, both of which were identified by Arksey and O’Malley [18] as the rationale for a scoping review [18]. This review followed the 6-stage methodology proposed by Levac et al [19]: (1) identifying the research question, (2) identifying all relevant publications, (3) selecting studies using inclusion and exclusion criteria, (4) charting the data to be extracted from each study, (5) synthesizing the data, and (6) reporting results. As the research questions of interest are described in the *Objectives* section, the *Methods* section will outline stages 2 to 5. The protocol for this scoping review was registered in Open Science Framework [20].

### Identifying Relevant Publications

We conducted an extensive search combining natural language and controlled vocabulary searching to capture the concepts of mHealth, app development, and nurses. We defined an mHealth app as a portable device that must interface with a patient or consumer and facilitate the information and data collection and delivery. It may interface with sensors, wearables, or cameras and may be connected to the internet. It includes health-related data and may assist with clinical decision-making. We defined app development as 1 of the first 5 phases of the Software Development Life Cycle (SDLC): planning, gathering requirements, design and prototyping, software development, and testing [21]. Planning includes allocating resources, scheduling the project, and determining costs. Requirements gathering engages subject matter experts (SMEs), technology specialists, and others to understand the necessary elements of the tool. Design and prototyping involve rapid preliminary prototyping to identify possible technical solutions. Software development advances the app from prototype to functional software. Finally, testing before deployment ensures that the app is fully operational and ready to be implemented in production. We chose to focus on these preimplementation phases of development, as nurses’ roles in implementation and adoption have been more fully explored in previous systematic reviews [22-24]. Health care providers included all licensed nurses, those in training to become nurses, subject matter nurse experts and health information technology nurse experts.

A librarian conducted the search across 14 databases: CINAHL via EBSCO, Cochrane via Wiley, Compendex via Engineering Village, Education Source via EBSCO, Embase via Ovid, ERIC via EBSCO, Global Index Medicus, Google Scholar, IEEE Xplore, MEDLINE via Ovid, PsycINFO via Ovid, PubMed, Scopus, and Web of Science Core Collection. The selected databases were chosen to represent an array of subject areas, including nursing, medicine, psychology, psychiatry, education, computer science, and engineering. Broad, multidisciplinary databases, such as Clarivate’s Web of Science Core Collection, Elsevier’s Scopus, and Google Scholar, were selected because of their coverage of nonarticle research outputs, such as conference papers and abstracts. A complete search strategy is presented in Multimedia Appendix 1. No restrictions were placed on the language, geography, or study design. The search was restricted to items published since 2016 to focus on the most recent developments in this field. The search was conducted in April 2021 and was updated in April 2023. To ensure that no potentially relevant studies were overlooked, we also hand searched the reference lists of the included publications. The results were compiled and deduplicated in EndNote (Clarivate).

### Selecting Studies

Items were first reviewed as titles and abstracts, followed by a full-text screening phase. A total of 2 independent researchers reviewed every item using Rayyan, a web-based tool that facilitates screening [25]. During the initial piloting phase, discrepancies were discussed as a group to establish a shared understanding of criteria and aims. Following this initial phase, a third researcher resolved discrepancies. We excluded publications that did not include an mHealth app, did not reference nurse involvement, did not include one of the relevant SDLC phases, or were published before 2016. We excluded review papers, including systematic reviews, and papers describing or reviewing previously developed apps. These exclusion criteria were used during the project’s title-abstract screening and full-text screening phases. Items retrieved through hand searching followed the same screening process and used the same inclusion and exclusion criteria. During the full-text screening phase, reasons for exclusion were recorded and reported in a PRISMA (Preferred Reporting Items for Systematic Reviews and Meta-Analyses) diagram.

### Charting the Data

A total of 2 reviewers extracted data from each article into REDCap (Research Electronic Data Capture; Vanderbilt University), a secure web-based data capture and management platform [26]. We used this platform to gather the following data: the SDLC phase or phases included in the publication, the role of the nurse in any of the phases, details regarding the app, and bibliographic details of the publication. Details regarding the app included the primary and secondary users, meaning the intended audience and other individuals who could engage with the app such as for data entry; the functionality of the app; the version of the app; and the condition or purpose of the app. The app’s functionality was identified using the IMS Institute for Healthcare Informatics functionality scoring system [27], a well-established scale for functionality assessment [28-30]. The IMS Institute for Healthcare Informatics functionality scoring system consists of 7 main categories and 4 subcategories, and the overall functionality score, between 0 and 11, is calculated by summing the scores across individual items, where 1 indicates presence and 0 indicates absence. Where the publication was in a language not spoken by the research team members, Google Translate was used to create an English-language translation, as Google Translate had been previously found to have a high level of accuracy when used to facilitate data extraction in evidence synthesis [31].

### Synthesizing the Data

After data extraction was completed, the findings were summarized according to the 3 research questions. Descriptive statistics were used to assess the frequency of roles, phases according to the SDLC, and the co-occurrence of specific roles and phases. The summary of the description of the apps included the IMS functionality, the condition of interest or purpose of the app, the primary and secondary users, the version of the app, and whether technical or content standards were used during the development of mHealth apps.

## Results

### Overview

**Figure 1:**
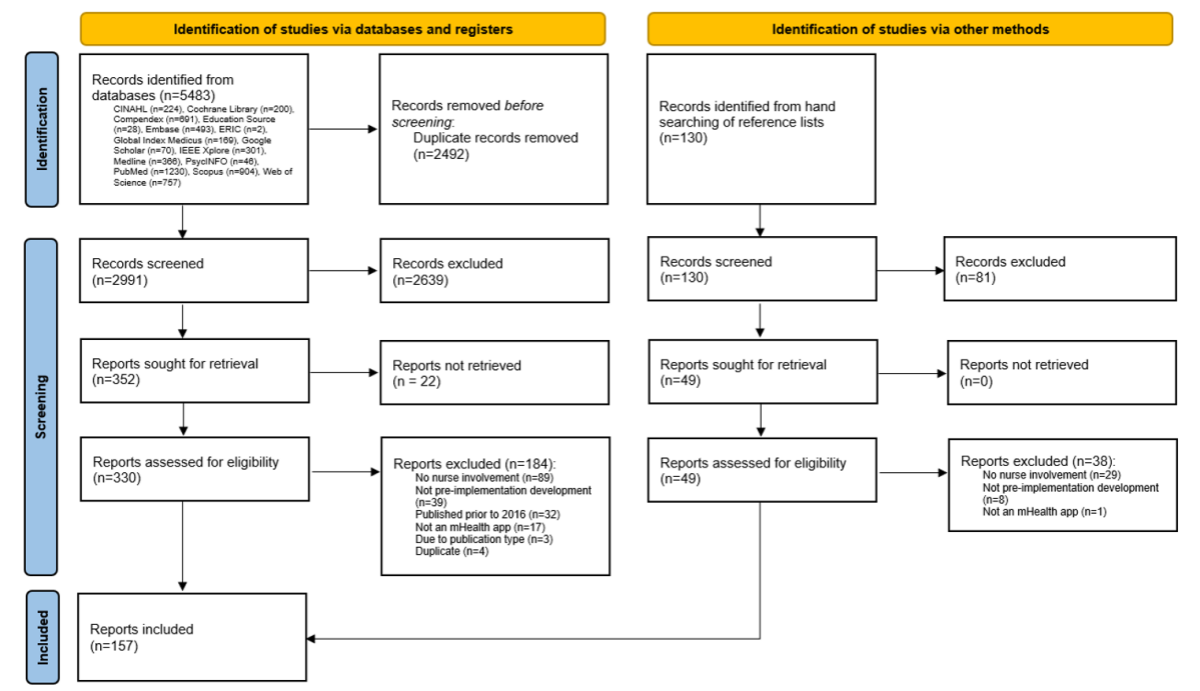
PRISMA (Preferred Reporting Items for Systematic Reviews and Meta-Analyses) flow diagram. mHealth: mobile health.

We retrieved 5483 items through database searching, 2492 (45.45%) of which were duplicates, resulting in 2991 (55.55%) items being screened at the title-abstract level. Following the title-abstract screening, 11.03% (330/2991) of the items were reviewed in full text, leading to 4.88% (146/2991) of publications that met the inclusion criteria [32-176]. Hand searching of reference lists identified additional 8.5% (11/130) of publications [177-187], resulting in 2.8% (157/5613) of included publications. There were 0.4% (22/5483) of items in the title-abstract screening phase that could not be retrieved for full-text screening through the resources available via any of the institutions or via interlibrary loans. Reasons for exclusion are presented in Figure 1.

The characteristics of the included studies are summarized in Table 1. Of the 157 included publications, 132 (84.1%) either described usability testing or were original research papers [32-49,51,52,55,56,58-65,67-75,77-81,83,85-87,89-107,110,112-117,119-124 ,126,129,130,132-134,136,138,140-145,147-160,162-169,171-173,175-187].

Of the 157 publications, 8 (5.1%) were conference abstracts [76,82,125,137,161,170,174,188], 7 (4.5%) were case reports [84,108,111,118,127,128,146], and 3 (1.9%) were commentaries or editorials [66,88,135]. The remaining publications (7/157, 4.5%) were protocols or descriptions of theoretical frameworks [50,53,54,57,109,130,139]. A total of 34 countries were represented in the publications, most frequently Brazil (29/157, 18.5%) [36,37,40,45,48,60,61,65,70,83,87,114,115,117,120, 134,136,138,142,143,152-154,157,163,165,177,182,183], the United States (26/157, 16.6%) [33,38,41,42,44,47,54, 57,66,68,69,71,76,81,89,97,102,103,107,113,121,132,160,164, 170,181], South Korea (10/157, 6.4%) [56,58,101,105, 118,133,149,156,180,184], and China (9/157, 5.7%) [90,95,110,127,167,172,173,176,186].

**Table 1:** Characteristics of the included studies.

Study, year	Publication type or study design (country)	Condition or purpose of app (primary user group or groups)	Development phase or phases described	Nurse’s role or roles during development	IMS Institute for Healthcare Informatics functionality score (0-11)
Abbasi et al [32], 2023	Usability testing (Iran)	Medication dosage or ICU^a^ (nurse)	Requirements and design and prototyping	Evaluator and informant or SME^b^	1
Achury Saldaña et al [145], 2021	Research (Colombia)	Heart failure (client or patient)	Design and prototyping and testing	Evaluator	5
Adib et al [33], 2022	Usability testing (United States)	Pediatric transplantation (caregiver)	Planning, requirements, design and prototyping, software development, and testing	Evaluator and informant or SME	7
Alexandrou et al [34], 2021	Research (Sweden)	Childhood obesity prevention (caregiver)	Requirements and design and prototyping	Evaluator and informant or SME	5
Alhodaib et al [35], 2020	Usability testing (United Kingdom)	Diabetes or chronic kidney disease (nurse and nonnurse provider)	Requirements, design and prototyping, and testing	Evaluator and informant or SME	7
Alves et al [36], 2021	Usability testing (Brazil)	Sexual violence care (nurse and nonnurse provider)	Requirements and design and prototyping	Evaluator and informant or SME	3
Alves et al [37], 2022	Usability testing (Brazil)	COVID-19 (nurse)	Requirements, design and prototyping, and testing	Evaluator and informant or SME	2
Anderson et al [38], 2021	Usability testing (United States)	Oncology (patient)	Testing	Research expert, patient advocate, and distributor	7
Andrades-González and Molina-Mula [39], 2022	Research (Spain)	Stroke (caregiver)	Requirements and design and prototyping	Evaluator, informant or SME, and research expert	2
Araujo et al [40], 2019	Usability testing (Brazil)	Neonatal care or NICU^c^ (nurse)	Requirements and testing	Evaluator and informant or SME	5
Aronson et al [41], 2021	Usability testing (United States)	Neonatal care (caregiver)	Requirements, design and prototyping, software development, and testing	Evaluator and informant or SME	5
Athilingam et al [42], 2016	Research or usability testing (United States)	Heart failure (client or patient)	Design and prototyping and software development	Content developer, designer or creator, evaluator, and research expert	2
Austin et al [43], 2022	Usability testing (Netherlands)	Oncology (client or patient)	Requirements, design and prototyping, and testing	Informant or SME, patient advocate, and evaluator	8
Awan et al [44], 2018	Usability testing (United States)	EHR^d^ documentation (nurse)	Testing	Evaluator	5
Barbosa de Lira et al [45], 2020	Research or usability testing (Brazil)	Older adults’ care (caregiver)	Design and prototyping and testing	Content developer, designer or creator, and evaluator	2
Barros et al [177], 2019	Research (Brazil)	Consciousness level assessment (student nurse)	Design and prototyping and testing	Evaluator and informant or SME	1
Benda et al [46], 2022	Usability testing (Myanmar)	Population health surveillance (nurse)	Requirements and design and prototyping	Evaluator and informant or SME	3
Berg et al [47], 2021	Research (United States)	Medication errors (nurse)	Testing	Research expert and evaluator	4
Bonifácio et al [48], 2021	Research (Brazil)	COVID-19 (client or patient)	Requirements	Evaluator	1
Bootsman et al [49], 2019	Usability testing (Netherlands)	Lower back posture (nurse and nonnurse provider)	Requirements, design and prototyping, software development, and testing	Evaluator and informant or SME	5
Børøsund et al [178], 2018	Research (Norway)	Mental health or oncology (client or patient)	Requirements, software development, and testing	Evaluator and informant or SME	7
Borycki et al [50], 2016	Theoretical framework (Canada, Australia, and Finland)	Nursing education (nurse, nonnurse provider, and student nurse)	Planning and requirements	Informant or SME	NR^e^
Broderick et al [188], 2016	Abstract (Ireland)	Pediatric hematology or oncology (caregiver and nurse)	Requirements, design and prototyping, software development, and testing	Content developer and informant or SME	2
Buinhas et al [51], 2019	Usability testing (Portugal)	Type 2 diabetes (client or patient)	Design and prototyping, software development, and testing	Evaluator and patient advocate	7
Calvillo-Arbizu et al [52], 2019	Usability testing (Spain)	Chronic kidney disease (caregiver, client or patient, nurse, and nonnurse provider)	Requirements, design and prototyping, software development, and testing	Evaluator and informant or SME	9
Castro et al [53], 2022	Protocol (Canada)	Palliative care or oncology (caregiver)	Requirements and design and prototyping	Evaluator and informant or SME	NR
Chalela et al [54], 2021	Protocol (United States)	Breast cancer (client or patient)	Requirements	Informant or SME	NR
Chávez et al [55], 2019	Usability testing (Mexico)	Alzheimer disease and dementia (nurse, nonnurse provider, and other)	Requirements, design and prototyping, and testing	Evaluator and informant or SME	4
Cho and Lee [56], 2017	Research or usability testing (South Korea)	Surgical safety (client or patient)	Requirements, design and prototyping, software development, and testing	Content developer, designer or creator, evaluator, informant or SME, distributor, and research expert	2
Choi et al [57], 2018	Theoretical model (United States)	Breast cancer or pain (client or patient)	Design and prototyping and testing	Content developer and designer or creator	4
Choi et al [58], 2021	Usability testing (South Korea)	Coronary artery disease (client or patient)	Requirements and design and prototyping	Content developer, evaluator, and informant or SME	6
Costa et al [59], 2021	Usability testing (Portugal)	Nursing home care (nurse and nonnurse provider)	Requirements	Informant or SME	7
Cruz et al [60], 2021	Usability testing (Brazil)	Breast cancer (client or patient)	Design and prototyping	Evaluator and informant or SME	5
da Silva et al [61], 2021	Usability testing (Brazil)	Blood donation (client or patient)	Requirements	Evaluator and informant or SME	4
de Dicastillo et al [62], 2019	Research or usability testing (Spain)	Surgery (client or patient and nurse)	Testing	Evaluator	5
de Dios et al [63], 2022	Usability testing (Spain)	HIV (client or patient)	Requirements and design and prototyping	Evaluator and informant or SME	7
de Jong et al [64], 2017	Research or usability testing (Netherlands)	Inflammatory bowel disease (client or patient, nurse, and nonnurse provider)	Requirements, design and prototyping, and testing	Evaluator and informant or SME	9
de Sousa et al [65], 2022	Usability testing (Brazil)	Heart failure (client or patient)	Design and prototyping	Evaluator	2
DeLemos [66], 2017	Briefs, commentaries, and editorials (United States)	Brain and nervous system diseases (nurse)	Software development	Not specified	3
Derksen et al [67], 2021	Usability testing (Netherlands)	Smoking cessation or pregnancy (client or patient)	Requirements and design and prototyping	Evaluator and informant or SME	5
Dodson and Layman [68], 2022	Usability testing (United States)	Pharmacogenomics or oncology (nurse)	Design and prototyping	Evaluator	4
Dodson and Layman [69], 2023	Usability testing (United States)	Pharmacogenetics (nurse)	Design and prototyping	Informant or SME	4
Duarte and Mandetta [70], 2022	Usability testing (Brazil)	Pediatric oncology or stem cell transplantation (caregiver and client or patient)	Design and prototyping	Evaluator	2
Durham et al [71], 2023	Research (United States)	Bladder exstrophy-epispadias-cloacal exstrophy complex (caregiver and client or patient)	Requirements	Research expert	2
Dürr et al [72], 2020	Usability testing (Germany)	Patient transfer safety (nurse)	Planning, requirements, and testing	Designer or creator, informant or SME, and evaluator	6
Ehrler et al [73], 2019	Research or usability testing (Switzerland)	Bedside care (nurse)	Requirements, design and prototyping, and testing	Evaluator and informant or SME	4
Ehrler et al [74], 2021	Research and usability testing (Switzerland)	Emergency department patient management (nurse and nonnurse provider)	Requirements and design and prototyping	Evaluator and informant or SME	3
Ekstedt et al [75], 2021	Usability testing (Sweden)	Chronic disease or older adults’ care (client or patient)	Requirements and design and prototyping	Evaluator and informant or SME	7
El-Jawahri et al [76], 2018	Abstract (United States)	Acute myeloid leukemia (client or patient)	Requirements and design and prototyping	Not specified	2
Elsayed Rashed et al [77], 2022	Research (Egypt)	EHR (student nurse)	Planning, requirements and design and prototyping	Evaluator and informant or SME	3
Elsbernd et al [78], 2018	Usability testing (Denmark)	Pediatric oncology (client or patient)	Planning, requirements and design and prototyping	Content developer and designer or creator	7
Escalada-Hernández [79], 2019	Usability testing (Spain)	Medical devices (nurse and nonnurse provider)	Requirements, design and prototyping, and software development	Evaluator and informant or SME	2
Esteves et al [80], 2019	Usability testing (Portugal)	Nursing home care (nurse and nonnurse provider)	Planning, requirements, and testing	Evaluator and informant or SME	5
Feldman et al [81], 2022	Usability testing (United States)	Immunizations or pediatric transplantation (caregiver and client or patient)	Planning, requirements, and design and prototyping	Informant or SME	6
Fernandez-Ortega et al [82], 2018	Abstract (Spain)	Chemotherapy-induced nausea and vomiting (client or patient and nurse)	Testing	Evaluator	NR
Ferreira et al [83], 2021	Usability testing (Brazil)	Nursing education (nurse)	Planning, requirements, and testing	Evaluator and informant or SME	2
Ferrua et al [84], 2020	Case report (France)	Oral cancer (client or patient and nurse)	Planning, requirements, and design and prototyping	Evaluator and informant or SME	9
Firdaus et al [85], 2022	Research and usability testing (Malaysia)	Diabetes (client or patient)	Planning and design and prototyping	Evaluator and informant or SME	2
Flohr et al [86], 2018	Usability testing (Canada)	Patient safety or ICU (nurse and nonnurse provider)	Planning, requirements, and design and prototyping	Evaluator and informant or SME	9
Franco et al [87], 2022	Research and usability testing (Brazil)	Pediatric oncology or medication management (client or patient)	Design and prototyping and testing	Evaluator	4
Fraser [88], 2018	Briefs, commentaries, and editorials (New Zealand)	Pediatric mental health (client or patient, nurse, and nonnurse provider)	Design and prototyping	Content developer, designer or creator, and evaluator	5
Gallimore et al [89], 2022	Usability testing (United States)	Urinary tract infections or COVID-19 (nurse)	Requirements	Informant or SME and research expert	6
Gao et al [90], 2020	Research or usability testing (China)	Pediatric mental health or pediatric oncology (caregiver, client or patient, nurse, and nonnurse provider)	Requirements, design and prototyping, and testing	Evaluator and informant or SME	5
Garne Holm et al [91], 2017	Usability testing (Denmark)	Neonatal care (caregiver, nurse, and nonnurse provider)	Requirements, design and prototyping, and testing	Evaluator and informant or SME	10
Given [92], 2017	Usability testing (Ireland)	Blood transfusion (nurse)	Planning, requirements, and design and prototyping	Content developer, designer or creator, and evaluator	6
Görtz et al [93], 2023	Usability testing (Germany)	Surgery (client or patient)	Design and prototyping	Evaluator and informant or SME	3
Grover et al [94], 2020	Usability testing (Botswana)	Oncology (nurse and nonnurse provider)	Testing	Evaluator	6
Guo et al [95], 2022	Research and usability testing (China)	Nutrition or macular degeneration (client or patient)	Requirements	Informant or SME	NR
Gutiérrez-Puertas et al [96], 2021	Research or usability testing (Spain)	Basic and advanced life support (student nurse)	Design and prototyping and testing	Evaluator	1
Harte et al [179], 2017	Research (Ireland)	Fall risk or older adults’ care (client or patient)	Testing	Evaluator	4
Herbert et al [97], 2021	Research (United States)	Heart failure (student nurse)	Testing	Evaluator	2
Hjorth-Johansen et al [98], 2022	Usability testing (Norway)	Severe cardiac disease or pediatrics (caregiver)	Requirements and testing	Evaluator and informant or SME	7
Hochstenbach et al [99], 2017	Usability testing (Netherlands)	Oncology or pain (client or patient and nurse)	Planning, requirements, and design and prototyping	Evaluator and informant or SME	11
Iyengar et al [100], 2021	Usability testing (Fiji)	Mental health (client or patient and nurse)	Design and prototyping and testing	Evaluator	3
Jeon et al [101], 2016	Research or usability testing (South Korea)	Metabolic syndrome (client or patient)	Requirements, design and prototyping, and testing	Content developer, designer or creator, and evaluator	10
Jones et al [102], 2017	Usability testing (United States)	Urinary tract infections (nurse and nonnurse provider)	Planning, requirements, design and prototyping, and testing	Evaluator and informant or SME	6
Keegan et al [103], 2016	Research (United States)	Neonatal care (student nurse)	Testing	Evaluator	2
Kho et al [104], 2019	Usability testing (Singapore)	Type 2 diabetes (client or patient)	Planning, requirements, design and prototyping, software development, and testing	Content developer, evaluator, and informant or SME	6
Kim et al [105], 2022	Usability testing (South Korea)	Peripheral artery disease (client or patient)	Requirements and testing	Evaluator and distributor	8
Kovach and Pollonini [107], 2022	Research (United States)	Pressure injuries (nurse)	Requirements and design and prototyping	Evaluator and informant or SME	6
Kurscheidt et al [108], 2022	Case report (Ireland)	Cystic fibrosis (client or patient)	Requirements	Informant or SME	8
Laranjeira et al [109], 2022	Protocol (Portugal)	Palliative care (caregiver and client or patient)	Requirements	Informant or SME	3
Lee and Kim [180], 2018	Research (South Korea)	Neonatal care or breastfeeding (client or patient)	Design and prototyping and testing	Evaluator	4
Lefco et al [181], 2017	Usability testing (United States)	Asthma (caregiver)	Requirements	Informant or SME	7
Liu et al [110], 2017	Usability testing (China)	Breast cancer (client or patient)	Planning, requirements, and design and prototyping	Content developer, designer or creator, evaluator, and informant or SME	1
Mallet et al [111], 2021	Case report (Canada)	Influenza (client or patient and nurse)	Design and prototyping	Evaluator	NR
Marino et al [112], 2022	Research (United Kingdom)	Nutrition or pediatrics (nurse and nonnurse provider)	Design and prototyping	Evaluator	6
Markossian et al [113], 2021	Usability testing (United States)	Chronic kidney disease (client or patient)	Design and prototyping	Evaluator and informant or SME	7
Melo et al [115], 2020	Usability testing (Brazil)	Nursing history and diagnosis (nurse and student nurse)	Design and prototyping	Evaluator	4
Miller et al [116], 2020	Usability testing (United Kingdom)	Colorectal cancer surgery (client or patient, nurse, and nonnurse provider)	Requirements and design and prototyping	Evaluator and informant or SME	10
Miranda and Salomé [117], 2022	Research (Brazil)	Pressure injuries (nurse and nonnurse provider)	Design and prototyping	Evaluator	6
Mohseni Moallem Kolaei et al [106], 2021	Usability testing (Iran)	Burns or delirium (nurse and nonnurse provider)	Planning, requirements, and design and prototyping	Evaluator and informant or SME	8
Moon et al [118], 2022	Case report (South Korea)	Delirium (nurse)	Requirements and design and prototyping	Evaluator	5
Morse et al [119], 2021	Usability testing (Tanzania)	Palliative care (client or patient, nurse, and nonnurse provider)	Requirements and testing	Evaluator and informant or SME	7
Motta et al [120], 2022	Usability testing (Brazil)	Cardio-respiratory arrest (student nurse)	Testing	Evaluator	2
Mueller et al [121], 2022	Usability testing (United States)	Pediatric oncology (caregiver)	Design and prototyping	Evaluator and informant or SME	5
Müller et al [122], 2021	Usability testing (Denmark)	Cardiopulmonary resuscitation (nurse and nonnurse provider)	Design and prototyping and testing	Evaluator	8
Muscat et al [123], 2021	Usability testing (Australia)	Chronic kidney disease (client or patient)	Planning, requirements, and design and prototyping	Content developer, designer or creator, evaluator, and informant or SME	3
Nes et al [124], 2023	Research (Norway)	Nursing education (nurse and student nurse)	Planning, requirements, and design and prototyping	Informant or SME	5
Neubeck et al [126], 2016	Usability testing (Australia)	Cardiovascular disease (client or patient)	Planning, requirements, and design and prototyping	Content developer, designer or creator, informant or SME, and informaticist	11
Neubeck et al [125], 2016	Abstract (Australia)	Cardiovascular disease (client or patient)	Planning, requirements, and design and prototyping	Content developer, designer or creator, informant or SME, and research expert	3
Ni et al [127], 2022	Case report (China)	Lung cancer (client or patient)	Design and prototyping	Evaluator and informant or SME	5
Noergaard et al [128], 2017	Case report (Denmark)	Heart disease (client or patient)	Design and prototyping, software development, and testing	Evaluator	9
Noori et al [129], 2016	Usability testing (Malaysia)	Mental health (caregiver and client or patient)	Requirements, design and prototyping, and testing	Content developer, evaluator and informant or SME	2
O’Connor and Andrews [130], 2016	Protocol (United Kingdom or Ireland)	Nursing education (student nurse)	Requirements and testing	Evaluator	4
O’Connor and Andrews [131], 2016	Usability testing (United Kingdom or Ireland)	Nursing education (student nurse)	Design and prototyping and testing	Designer or creator and evaluator	1
Odom and Christenbery [132], 2016	Usability testing (United States)	Asthma (client or patient)	Requirements, design and prototyping, and testing	Content developer, informant or SME, patient advocate, distributor, and research expert	3
Park and Cho [133], 2022	Research (South Korea)	Neonatal care (caregiver)	Design and prototyping and testing	Evaluator and informant or SME	4
Paschoal et al [134], 2022	Research (Brazil)	Diagnostic reasoning (nurse)	Planning, requirements, and design and prototyping	Designer or creator, evaluator, and informant or SME	2
Patel et al [135], 2019	Briefs, commentaries, and editorials (United Kingdom)	Wound care (nurse and nonnurse provider)	Requirements, design and prototyping, and testing	Content developer, designer or creator, evaluator, and distributor	7
Pereira et al [136], 2016	Usability testing (Brazil)	Vital Signs (student nurse)	Planning, requirements, design and prototyping, and software development	Content developer, designer or creator, and informant or SME	2
Pereira et al [182], 2019	Usability testing (Brazil)	Surgery (student nurse)	Design and prototyping	Evaluator	2
Pérez-Sádaba et al [137], 2022	Abstract (Spain)	Frailty (client or patient)	Requirements	Informant or SME	3
Pontes et al [138], 2021	Research (Brazil)	Clinical assessment of patients who are hospitalized (nurse)	Requirements and design and prototyping	Evaluator and informant or SME	4
Putri et al [139], 2022	Protocol (Indonesia)	Type 2 diabetes (client or patient)	Planning, design and prototyping, and testing	Evaluator, informant or SME, and research expert	NR
Ramli et al [140], 2022	Research (Malaysia)	Clinical management (client or patient)	Testing	Evaluator	6
Rathnayake et al [141], 2021	Research (Australia)	Dementia (caregiver)	Design and prototyping	Evaluator, informant or SME, and research expert	5
Rezende et al [142], 2016	Research or usability testing (Brazil)	NICU care (nurse)	Planning, design and prototyping, and testing	Evaluator and informaticist	4
Rutz et al [144], 2019	Research or usability testing (Germany)	Dementia (caregiver)	Planning and design and prototyping	Not specified	NR
Salomé et al [183], 2022	Usability testing (Brazil)	Upper airway aspiration (nurse and nonnurse provider)	Testing	Evaluator	5
Saparamadu et al [146], 2021	Case report (Singapore)	Delivery of lab information (nurse and other)	Requirements, design and prototyping, and testing	Designer or creator, evaluator, and informant or SME	3
Schmidt et al [147], 2021	Research (Germany)	Chewing efficiency (nurse)	Testing	Evaluator	8
Schweers et al [148], 2016	Usability testing (India)	Complications in childbirth (nurse and other)	Requirements and design and prototyping	Evaluator and informant or SME	6
Seo et al [184], 2021	Research (South Korea)	Postpartum depression (client or patient)	Requirements, design and prototyping, and testing	Content developer, evaluator, and informant or SME	8
Seok and Suh [149], 2022	Research and usability testing (South Korea)	Oncology (student nurse)	Design and prototyping and testing	Evaluator and informant or SME	6
Shahmoradi et al [150], 2021	Research and usability testing (Iran)	Urinary tract infections (client or patient)	Requirements and testing	Evaluator and informant or SME	7
Shahmoradi et al [151], 2021	Research (Iran)	Pain management (student nurse)	Requirements, design and prototyping, and testing	Content developer, designer or creator, evaluator, and informant or SME	5
da Silva Lima Roque et al [143], 2021	Usability testing (Brazil)	Wound care (nurse and nonnurse provider)	Software development and testing	Evaluator	5
da Silva Melo et al [114], 2020	Usability testing (Brazil)	Diabetes (client or patient and nurse)	Requirements, design and prototyping, software development, and testing	Content developer, evaluator, and informant or SME	9
Silva et al [153], 2021	Usability testing (Brazil)	Domestic violence against children (nurse)	Planning, requirements, and design and prototyping	Content developer, designer or creator, and informant or SME	5
Silva et al [152], 2022	Research (Brazil)	Pregnancy and postnatal care (client or patient)	Planning, design and prototyping, and testing	Designer or creator, evaluator, and informant or SME	4
Sobrinho et al [154], 2018	Research or usability testing (Brazil)	Chronic kidney disease (client or patient)	Requirements	Informant or SME	9
Soilemezi et al [155], 2021	Research (Greece)	Hemodialysis patient handoff (nurse)	Planning, requirements, design and prototyping, software development, and testing	Evaluator and informant or SME	5
Song and An [156], 2021	Usability testing (South Korea)	Chronic myeloid leukemia (client or patient)	Testing	Informant or SME	6
Souza et al [157], 2022	Research (Brazil)	Prenatal care (client or patient)	Design and prototyping and testing	Evaluator and informant or SME	7
Strandell-Laine et al [158], 2019	Research (Finland)	Nursing education (nurse and student nurse)	Requirements, design and prototyping, software development, and testing	Content developer, evaluator, and informant or SME	4
Sun et al [159], 2021	Research (United Kingdom)	Esophageal cancer (caregiver and client or patient)	Requirements and testing	Evaluator and informant or SME	6
Sundaram et al [160], 2023	Research (United States)	Spinal cord injury (client or patient)	Planning, requirements, design and prototyping, and testing	Evaluator and informant or SME	6
Tamamoto et al [161], 2017	Abstract (Japan)	Chronic obstructive pulmonary disease (client or patient)	Design and prototyping	Not specified	3
Tan et al [162], 2023	Research (Singapore)	COVID-19 (client or patient)	Requirements, design and prototyping, and testing	Designer or creator, evaluator, and informant or SME	7
Torrente et al [163], 2021	Research (Brazil)	Emergency services (client or patient)	Requirements, design and prototyping, and software development	Designer or creator and informant or SME	5
Vamos et al [164], 2019	Research or usability testing (United States)	Oral Health or pregnancy (nurse)	Requirements, design and prototyping, and testing	Designer or creator, evaluator, and informant or SME	6
Vêscovi et al [165], 2017	Usability testing (Brazil)	Diabetes or foot care (nurse)	Requirements, design and prototyping, and testing	Content developer, designer or creator, evaluator, and informant or SME	4
Vilarinho et al [185], 2017	Research (Norway)	Cystic fibrosis (client or patient)	Requirements, and design and prototyping	Evaluator and informant or SME	5
Wan et al [166], 2021	Research (Singapore)	Colorectal cancer (caregiver, client, or patient)	Requirements	Designer or creator and informant or SME	3
Wang et al [167], 2017	Usability testing (China)	Pediatrics (caregiver, client or patient, and nurse)	Planning, requirements, design and prototyping, and testing	Evaluator and informant or SME	4
Wang [168], 2017	Research (Singapore)	Type 2 diabetes (client or patient)	Requirements and testing	Content developer, evaluator, informant or SME, and research expert	7
Wang et al [186], 2021	Research (China)	Lung cancer (client or patient)	Requirements and design and prototyping	Evaluator and informant or SME	6
Wannheden and Revenäs [169], 2020	Usability testing (Sweden)	Parkinson disease (client or patient, nurse, and nonnurse provider)	Planning, requirements, design and prototyping, and testing	Designer or creator, evaluator, and informant or SME	9
Wirawan and Arsa [187], 2020	Research (Indonesia)	Basic life support or cardiac arrest (nurse and nonnurse provider)	Requirements	Informant or SME	2
Woo et al [170], 2016	Abstract (United States)	Medication management (client or patient and nonnurse provider)	Planning and requirements	Informant or SME	3
Woods et al [171], 2018	Usability testing (Australia)	Heart failure (caregiver and client or patient)	Requirements and design and prototyping	Designer or creator, evaluator, and informant or SME	NR
Yang et al [172], 2016	Research or usability testing (China)	Delirium or ICU (nurse)	Software development and testing	Evaluator	5
Yang et al [173], 2022	Research (China)	Chronic disease (client or patient)	Requirements and design and prototyping	Content developer, designer or creator, informant or SME, and research expert	5
Yerlett et al [174], 2021	Abstract (United Kingdom)	Epidermolysis bullosa (client or patient)	Design and prototyping	Designer or creator and informant or SME	3
Ying et al [175], 2022	Research (Singapore)	Care coordination (nurse and nonnurse provider)	Design and prototyping and testing	Designer or creator, evaluator, and informant or SME	5
Zhang et al [176], 2021	Research (China)	Wound care (nurse)	Design and prototyping and testing	Evaluator	7

^a^ICU: intensive care unit.

^b^SME: subject matter expert.

^c^NICU: neonatal intensive care unit.

^d^EHR: electronic health record.

^e^NR: not reported.

### Nurse Involvement

Nurses played a role in all the stages of development. Nurses were most represented in the design and prototyping phase (112/157, 71.3%) [32-37,39,41-43,45,46,49,51-53,55-58, 60,63-65,67-70,73-79,81,84-88,90-93,96,99-102,104,106,107,110-118, 121-129,131-136,138,139,141,142,144-146,148,149,151-153,155, 157,158,160-165,167,169,171,173-177,180,182,184-186,188], followed by the requirements gathering phase (98/157, 62.4%) [32-37,39-41,43,46,48-50,52-54,56,58,59,61,63,64,67,71-81,83, 84,86,89-92,95,98,99,101,102,104-110,114,116,118,119, 123-126,129,130,132,134-138,146,148,150,151,153-155,158-160, 162-171,173,178,181,184-188] and the testing phase (80/157, 51%) [33,35,37,38,40,41,43-45,47,49,51,52,55-57,62,64,72,73, 80,82,83,87,90,91,94,96-98,100-105,114,119,120, 122,128-133,135,139,140,142,143,145-147, 149-152,155-160,162,164,165,167-169,172,175-180,183,184,188]. Nurses were infrequently involved in the software development phase (20/157, 12.7%) [33,41,42,49,51,52,56,66,79,104, 114,128,136,143,155,158,163,172,178,188] and the planning phase (33/157, 21%) [33,50,72,77,78,80,81,83-86,92, 99,102,104,106,110,123-126,134,136,139,142,144,152,153,155,160,167,169,170].

Nurses most frequently assumed the role of evaluator, with 123 (78.3%) of 157 publications indicating that nurses took on this role in at least 1 phase [32-37,39-49,51-53,55,56,58, 60-65,67,68,70,72-75,77,79,80,82-88, 90-94,96-107,110-123,127-131,133-135,138-143,145-152,155, 157-160,162,164,165,167-169,171,172,175-180,182-186].

This was followed by the role of informant or SME (106/157, 67.5%) [32-37,39-41,43,46,49,50,52-56,58-61,63,64,67,69,72- 75,77,79-81,83-86,89-91,93,95,98,99,102, 104,106-110,113,114,116,119,121,123- 127,129,132-134,137-139,141,146,148-160, 162-171,173-175,177,178,181,182,184-188], designer or creator (31/157, 19.7%) [42,45,56,57,72,78,88,92,101,110,123,125,126, 131,134-136,146,151-153,162-166,169,171,173-175], or content developer (27/157, 17.2%) [42,45,56-58,78,88,92,101, 104,110,114,123,125,126,129,132, 135,136,151,153,158,165,168,173,184,188].

Nurses served in different roles across different phases of development. The most common co-occurrence was a nurse acting as an informant or SME during the requirements gathering phase (88/157, 56.1%) [32-37,39-41,43,46,49,50,52-56,58,59,61,63,64,67,72-75, 77,79-81,83,84,86,89-91,95,98,99,102, 104,106-110,114,116,119,123-126,129,132,134,136-138,146,148,150, 153-155,158-160,162-171,173,178,181,184-188], closely followed by acting as an evaluator during the design and prototyping phase (84/157, 53.5%) [32-34,36,37,39,41, 43,45,46,49,51-53,55,56,58,60,63-65,67,68,70,73-75,77, 79,84-88,90-93,96,99-102,104,106,107,111-118,121-123, 127,129,131,133-135,138-142,146,148,149,151, 157,158,164,165,167,169,171,180,182,184-186] or acting as an evaluator during the testing phase (71/157, 45.2%) [35,37,40,41,43-45,47,49,51,52,55,62,72,73,80,82,83,87, 90,91,94,96-98,100-105,114,119,120,122,128-130,133,135,139,140,142, 143,145-147,149-152,155,157-160, 162,164,165,167-169,172,175-180,183,184].

Roles were not mutually exclusive in the various development phases. The authors indicated that nurses took on between 1 and 4 roles within a development phase and more frequently took on a greater number of roles during earlier development phases. In total, 36% (12/33) of the publications describing the planning phase indicated nurses taking on >1 role [85,92,102,104,110,123,125,126,134,136,139,152]. Nurses taking on >1 role grew to 42 (43%) out of 98 publications in the requirements gathering phase [36,37,39,52,55,56,58,61,78,83,84,86,89,92, 99,101,104,110, 114,116,123,125,126,129,132,134,136,139,146,151,153,158,164-169,171,173,184,188] and 50 (44.6%) out of 112 publications in the design and prototyping phase [34,36,37,39,42,43,45,46,51,56-58,60,75,78, 88,92,93,101,104,110,113,114,121,123,125-127,131-136,138,141,142,146,151, 157,158,162-164,169,171,173-175,184]. None of the nurses took on >1 role during the software development phase, whereas 12 (15%) out of 80 publications noted nurses taking >1 role during the testing phase [38,43,47,51,56,132,135,139,149, 151,152,160].

### Characteristics of the Apps

Nurses were equally involved in developing apps for care providers and apps for health care consumers. Care providers were most frequently nurses (69/157, 43.9%) [32,35-37,40,44,46,47,49,50,52,55,59,62,64,66,68,69,72-74,79, 80,82-84,86,88-92,94,99,100,102,106,107,111,112,114-119, 122,124,134,135,138,142,143,146-148,153,155,158,164,165,167,169,172,175,176,183,187,188], followed by nonnurse providers (30/157, 19.1%) [35,36,49,50,52,55,59,64,74,79,80,86,88,90,91,94,102, 106,112,116,117,119,122,135,143,169,170,175,183,187], and student nurses (16/157, 10.2%) [50,77,96,97,103,115,120,124,130,131,136,149,151,158,177,182]. In 49.7% (78/157) of the publications, clients or patients were included as primary end users [38,42,43,48,51,52,54,56-58,60-65,67,70,71,75,76,78,81, 82,84,85,87,88,90,93,95,99-101,104,105,108-111,113,116,119,123,125-129, 132,137,139,140,145,150,152,154,156,157,159-163,166-171,173,174,178-180,184-186]; in 15.9% (25/157) of the studies, caregivers were considered primary end users [33,34,39,41,45,52,53,70,71,81,90,91, 98,109,121,129,133,141,144,159,166,167,171,181,188]. This pattern continued when considering secondary end users, which we defined as individuals interacting with the app to enter data but who may not be the intended market for the app. Although care providers were more prominent than health care consumers as secondary end users, both were represented. Nurses were secondary end users in 57.3% (90/157) of the publications [32-35,38,40,41,44,49,52-55,61-64,66,67,71-75,79-84,86-88, 90-92,94,98-100,102,104-109,111,113-116,119-122,128,129,132,135,137,139- 142,145,146,148,150,152-154,157,158,160,162-170,172,173,176,179,184,185], whereas nonnurse providers were noted as secondary end users in 42.7% (67/157) of the publications [35,38,41,46,49,52-55,61,63,64,68,69,71,72,74, 79-81,86-88,90,91,94,98,102,105,106,108,109,113,116,118-123,128,129,132,135,137,139- 141,145,147,150,152,154,157,160,162,163,166,167,169,170,172,173,175,179,184,185], and clients or patients were noted as secondary end users in 26.8% (42/157) of the publications [42,49,51,52,56-58,60,62,64,76, 78,82,84,85,88,90,93,95,99,101,104,106,110,111, 114,116,119,123,125,126,128,129,132,143,154,161,167-171].

In general, the apps focused on specific conditions, most frequently cancer (27/157, 17.2%) [38,43,53,54,57,60,68,70,76,78,82,84,87,90,94, 99,110,116,127,149,156,159,166,178,186,188, 189], cardiovascular disease (12/157, 7.6%) [42,58,65,97,98,120,125,126,128,145,171,187], pregnancy or neonatal care (11/157, 7%) [40,41,67,91,103,133,152,157,164,180,184], and diabetes (8/157, 5.1%) [35,51,85,104,114,139,165,168]. These apps were often in the early stages of development, although 26.1% (41/157) of the publications described multiple versions of the app [33,34,41-43,57,58,63,71,73,77,78,83,86,92, 95,99,102,104, 116,119,122,123,126,128,129,135,136,145,146,148,153,158-160,171,172,175,177,178,184]. Most commonly, the apps were the alpha or prototype versions (117/157, 74.5%) [32,33,35,37,39-43,45,46,49, 51,52,55-58,60,61,63,65,67,68,71,73-75,77-80,83-88,90-93,95, 98-107,110-119,121-126,128,129,131,132,134-136,138,140-143,146-167,169-181,184], followed by the storyboard or wireframe (32/157, 20.4%) [33,41,43,48,57,59,70,71,76,78,81,83,86,92,102,108,116,122,123, 126,128,129,136,146,148,153,158,171,177,178,185,186], beta (29/157, 18.5%) [33,34,36,38,42,43,47,58,63,69,71-73,77, 89,95,97,99,104,119,133,135,145,159,160,168,172,175,184], and release (10/157, 6.4%) [33,34,62,66,71,94,120,135,145,188] versions. In total, 10.2% (16/157) of the publications did not report the version of the app or described apps that had not yet been created [44,50,53,54,64,82,96,109,127,130,137,139, 144,182,183,187].

The median number of functions based on the IMS Institute for Healthcare Informatics Functionality was 5 (range 1-11). A total of 2 apps scored 11, the maximum possible score on the IMS Institute for Healthcare Informatics Functionality score [99,126]. Most publications (142/157, 90.4%) described multifunctional apps, with only 15 (9.6%) out of 157 publications describing an app with only a single function. The most frequently reported functionalities were to inform (101/157, 64.3%) [34-37,39,41-43,45,48,49,56,57,59-61,63-67,70-72,75,76,78,79,81, 83-88,90,91,93,97-99,101,103-110,113-116,120,122,123,125-130,132-136, 138,140,141,146,147,149-151,153,154,157,159-166,168, 173,174,176-178,180-184,186-188], collect data (100/157, 63.7%) [33,35,38,43,44,46,47,49,51,52,55,57-64,67-69,72,73, 75,77,78,80,81,84,86-92,94,98-102,104-106,108,112-114, 116-119,121,122,124,126-128,130,132,133,135,137,138, 140-143,145,147-150,152-159, 162-164,167-169,172,173,175,176,178,179,181,183-186], or instruct (80/157, 51%) [33-37,39,40,42,43,45,56,57,60,64-67,70-72,75, 76,78,79,85,91,93,97-99,101-104,106-109,114,115,120,122,123,125-131,134-136,141, 143,146,147,149-151,154,157,160,161,164-166,168,170, 173,174,178,180-184,186-188]. The least frequently reported functionalities were to intervene (29/157, 18.5%) [33,38,40,49,51,52,59,68,84,86, 89,92,99,101,107,116-118,122,126,128,147,148,153,154,156,169,178,181], remind or alert (41/157, 26.1%) [33,38, 43,44,52,59,60,63, 72,75,81,84,87,91,94,99,101,105,107-109,113,116,126,140,145,146,148,150,157,159,160, 162,166,168,169, 179,180,184,185,189], and evaluate data (48/157, 30.6%) [35,43,44,47,51,52,55,58,62,64,80, 84,86,89,91,92,94,98,99,101,102,106,112-114,116-119,122,126,128,135,142, 143,147,148,151,153,154,165,167,169,170,172,176,184,186]. Some of the apps integrated with other tools, with a majority integrating with some form of communication (39/157, 24.8%) [34,38,44,51,54,58,60,64,67,71,74,75,81,83,84,86,88,91,94,99, 106,119,126,128,133,138,143, 145,148,152,154,157-160,169,180,184,186], external resources (30/157, 19.1%) [32,34,36-39,43,48,54,58,60,61,71,75,81,83,84,89, 105,111,118,123,126,128,133,135,145,146,159,184], or electronic health record (21/157, 13.4%) [38,44,47,58,59,73,77,81, 84,88,91,126,132,135,145,152,159,164,167,169,175]. Apps less frequently integrated with portals (15/157, 9.6%) [38,52,59-63,84,86,99,105,118,119, 126,145], sensors or add-ons (8/157, 5.1%) [38,49,62,72,86,105,160,179], or other tools (ie, cloud-based servers and storage or databases; 5/157, 3.2%) [33,103,141,172,176]. Many apps (85/157, 54.1%) did not report integration with other devices.

The use of technical standards and standards for guiding content was also variable. In total, 26.8% (42/157) of the publications used ≥1 standard. In total, 8.9% (14/157) of the publications included content standards [41,96,97,102,127,153,164,167,177,181,182,184,186,187], such as clinical practice guidelines or recommendations for patient care, whereas 14% (22/157) included technical standards that informed the app’s development [37,40,52,59,60,67,75,88,104,108,114,115,117,123,134,151,159,165,172,173,178, 179], and 3.8% (6/157) of the publications included both technical and content standards [36,68,69,145,154,158]. Most of the manuscripts (115/157, 73.2%) did not indicate using a standard when developing their app.

## Discussion

### Principal Findings

This scoping review illuminated the various roles of nurses in mHealth app development. However, considering the number of published materials, few publications describe the development process of mHealth apps. This review reinforces previous findings that the levels of overall provider representation in app development are low, despite the recognition that provider involvement is key to creating effective apps [190-193]. Although nurses were represented throughout the development process in this review, the level of involvement was concentrated in specific phases and roles. Nurses were most frequently involved in the requirements gathering, designing and prototyping, and testing phases of development. Despite established recommendations for co-design and participatory design in mHealth, which reinforce the importance of involving clinicians and patients early and throughout the process [194,195], we found more limited involvement of nurses in the planning and software development phases. This parallels the findings of a recent rapid review on co-design practices in mHealth, which found that both patients and health care providers were most frequently engaged in needs identification, prototype design, and feedback and testing [196].

We found that the most common role for a nurse was an SME during the requirements gathering phase, followed by an evaluator during the testing phase. However, the roles for nurses as research experts, patient advocates, or informaticists were rare. This concentration in specific phases and roles reinforces the previous findings. A systematic review of clinician involvement in developing predictive clinical decision support tools found that clinicians most frequently served as informants (specifically, identifiers of system needs and requirements and developing clinically relevant content) but were less involved in evaluation [197]. Another systematic review of pain management apps found that most apps included in their study indicated that a health care professional was involved in the development, which was limited to content development or the role of a SME [198]. Although our study found that nurses were involved in content development and served as SMEs, involvement in testing and evaluation was prominent. The role of patient advocate may be implicit given the patient-centered focus of nurses; however, the role was rarely named as such. Although not named as patient advocates, nurses were frequently involved in the development of apps that included patients or caregivers as primary end users. This may be in recognition of the nurses’ understanding of patient needs concerning their health, diagnoses, and treatment options, given that nurses spend time and interact with patients more than any other health care provider. Patients are most likely to adopt an mHealth app when they believe that the information and services provided are trustworthy [199,200], and previous systematic reviews have found a strong, positive relationship between a patient’s perception of the opinions of those important to them and the intention to use mHealth apps [199,201]. In 1 study of older adults, 64% either agreed or strongly agreed that they would use an mHealth app if their health care provider recommended it [202]. A systematic review of barriers and facilitators in mHealth in oncology noted that “[n]urses seemed to the stakeholders with the greatest potential to push mHealth uptake” [203].

In the case of patient-facing solutions, nurses often have a unique perspective as patient advocates. For example, nurses can routinely answer questions from patients and families related to the use of apps or their health data [189,204]. Leveraging first-hand accounts of these experiences can provide important insights into how an app may be best designed for optimal patient engagement and ease of use [205]. Nurse-led and nurse-supported mHealth interventions have been associated with increased patient compliance, more complete self-reporting, and healthier lifestyle choices [206,207]. We believe that nurses should play an integral role in developing apps intended for patient or caregiver use and that their role as patient advocates should be integrated throughout the development process.

Nurses frequently assumed the role of evaluator throughout the development process. Although nurses responded to information, decisions, or app functionality, they were less frequently tasked with developing the app or designing its content. Nurses are more frequently evaluating or informing, rather than designing and developing, which may reflect previous findings that nurses have had limited involvement in mHealth app development [204]. The apps described in this scoping review lack robust functionality such as facilitating communication between systems and health care teams and evaluating data entered by the end user. These findings are not surprising because apps are relatively new to health care systems [208]. Our results partially parallel the IMS Institute’s findings, in which the authors assessed 23,682 apps to determine their functionality according to the IMS Institute for Healthcare Informatics Functionality. They found that the most widely available individual functionality was to inform, followed by instruct, and noted that most apps supported only a single functionality rather than being multifunctional. The authors noted that “most of the healthcare apps available today are only simple in design and do little more than provide information” [27]. Most apps included in our study were multifunctional, unlike those evaluated by the IMS Institute. However, the most frequent functionalities were focused on providing information to users. Although some more advanced functionalities were available in the apps included in our study, such as facilitating bidirectional communication with health care providers or providing reminders or alerts to patients, this was less common.

Previous systematic reviews have noted that apps have limited clinical utility and do not facilitate intervention or adjustment of care plans; instead, they focus on collecting patient data or providing basic information [191]. As apps become more sophisticated with sensor technology and interoperable with clinical decision support systems, adopting mHealth standards to support more advanced functions and features will be essential. Standards for mHealth app development exist but are rarely described in the literature, with the vast majority of publications lacking a description of technical standards or content standards. The lack of documentation describing the evidence base underlying these apps is a glaring omission and, unfortunately, is consistent with the findings of previous research [192,209,210]. Equally important to content standards are app development standards to ensure interoperability with other systems, usability, effective data capture and transfer, and the protection of patient information.

Further complicating the disorganized approach to mHealth development is an absent organizing body to establish and manage standards, ways to update and maintain systems, an overarching interoperable plan in health care, and the enterprising nature of technology. Those who develop technologies, including technologies for health care, are business oriented with a budget and prioritize profits over collaboration. Instead, businesses prefer to have more clients and purchasers than to become interoperable with competing systems.

To overcome some of these barriers, nurses have a role in planning and developing mHealth apps. Interoperable systems that support data sharing and transfer strengthen the provision of quality care to patients, who are now more involved in their care and decision-making. Creating these systems requires individuals with a knowledge base of institutional systems and health care as well as working knowledge of the fundamental needs of patients. These characteristics define a nurse informaticist. A nurse informaticist offers a clinical perspective combined with expertise in the technological systems and structures underlying patient care. Nurse informaticists are uniquely qualified because they have expertise in the health care environment and technology, functioning as liaisons between the clinical team, patients, and developers. The nurse informaticist transforms data into information and knowledge to be leveraged by technologies within a given environment to improve health, health care equity, safety, quality, and outcomes [211].

It is not surprising that apps focusing on cancer were the most prevalent in publications included in our review. Oncology has been recognized as a key space for nurse informaticists because of the complexity of coordinating care across multiple specialties and settings and the subsequent range of eHealth tools that have emerged as a result of this complexity [212-214]. Although cancer was the most predominant condition, many of the apps included in this review focused on complex, chronic conditions, such as cardiovascular diseases, diabetes, chronic kidney disease, and mental health. Chronic conditions have been previously identified as an opportunity for mHealth, given that the management of chronic conditions requires frequent data collection and transfer better suited to mHealth interventions than conventional office visits [215,216]. Patient engagement is key to the success of mHealth interventions for managing of chronic conditions. As previously noted, nurses can have a pivotal role in both motivating patients to start using the app and providing ongoing support and guidance for its continued use.

mHealth apps are still in their infancy, as is the nurses’ role in these apps. Nurses have a depth and breadth of knowledge in the health care environment, disease management, organizational infrastructure, quality, safety, patient education, and evidence-based practice. As McGonigle and Mastrian [217] note, “nursing is an information-intensive profession,” and “[a]t the heart of all of these [patient care] skills lies the management of data and information.” The accuracy of the information and the guidance an app may offer cannot be validated without due diligence by the health care professionals and patients. One way to ensure that health-related content is valid, based on evidence, and meets the needs of the end user is to incorporate nurses into the early stages of app creation [204]. Interventions fail most frequently because of misalignment between the app and existing workflows and clinical processes, leading to increased workloads or cumbersome workflows [218]. The technological burden for clinicians is at an all-time high. This technological stress contributes to clinician burnout, resulting in clinicians leaving the workforce [219,220]. Including nurses in the development of technologies can provide a sense of value while simultaneously increasing the likelihood of successful implementation by addressing workflow and clinical misalignment issues during the design process.

### Limitations and Future Work

The full spectrum of app development efforts might not be fully documented or reported in the published literature. Although this review included conference presentations, abstracts, and protocol papers to capture ongoing projects and projects communicated outside of research articles, there may be previous or ongoing development efforts in which nurses are involved that are described only in internal documents or materials restricted owing to intellectual property considerations. Although we developed a search strategy in accordance with best practices and attempted to be as comprehensive as possible, our search terms may not include relevant terminology in every discipline. Despite this, we believe that we have captured all publications meeting our inclusion criteria.

Our decision to include protocol papers means that some description of nurse involvement and the phase of involvement is anticipated rather than having been completed. Although it is possible that projects ultimately deviate from their published protocols, we believe that these descriptions of planned involvement, nevertheless, contribute to addressing our overall research questions.

Our review is limited to examining the reported role of nurses in mHealth app development and does not account for the postimplementation involvement of nurses. Our analysis is also limited to what is described in the publication. It is possible that the nurses took on additional roles or were engaged in other phases that were not described in the publication. Future research should consider additional research methods, such as surveys, to understand the role and experience of nurses in mHealth app development beyond what is documented in the literature. Similarly, it is possible that technical and content standards were used more broadly, but their use was not described in publication. More complete reporting of standards would more effectively communicate which standards are most broadly implemented and subsequently promote further awareness and adoption.

This scoping review did not examine the efficacy of mHealth apps or their impact on clinically relevant outcomes. As more randomized controlled trials become available, future research may consider quantifying the impact of nurse involvement in mHealth app development on clinical outcomes.

Although we did not assess the risk of bias in the included studies, as this is not a component of scoping reviews, there is a lack of clarity regarding funding sources. Depending on the funding models for app development, there may be a conflict of interest on the part of the authors describing the app development process or its outcomes. This lack of information makes it difficult to assess whether any conflict of interest existed. Although the potential conflict of interest may not directly affect our findings, clear reporting of funding sources and any conflicts of interest enables a more thorough examination of the quality of the research.

### Conclusions

Regardless of the type of health system, nurses must work toward nurse representation on all technology-specific committees and task forces influencing health care services. Currently, the role of nurses in mHealth app development is limited, although the potential benefit of incorporating this expertise throughout the development process would be to patients, providers, and care systems. Advocacy for nurse involvement in planning, development, implementation, and evaluation is a vital role for nursing leadership in all care systems, and widespread communication and dissemination of these roles can serve as an example for those developing mHealth apps.

## Appendix

Multimedia Appendix 1 Search strategies.

Multimedia Appendix 2 PRISMA-ScR checklist.

## Abbreviations

mHealth: mobile health
PRISMA: Preferred Reporting Items for Systematic Reviews and Meta-Analyses
REDCap: Research Electronic Data Capture
SDLC: Software Development Life Cycle
SME: subject matter expert
