# Supplementary material for: Nurses’ Roles in mHealth App Development: Scoping Review
Source: JMIR Nurs. 2023 Oct 17;6:e46058. doi: 10.2196/46058 (PMC10618897; doi:10.2196/46058)
Supplement: Multimedia Appendix 1 [file nursing_v6i1e46058_app1.pdf]

Supplement: Search Strategies

CINAHL via EBSCO

1. (MH "Telehealth+")
2. TI(mobile OR app OR apps OR application*) OR AB(mobile OR app OR apps OR application*)
3. S1 AND S2
4. (MH "Cellular Phone+")
5. TI(app OR apps OR application*) OR AB(app OR apps OR application*)
6. S4 AND S5
7. TI((mobile OR smartphone* OR "cell phone*" OR cellphone*) N3 (app OR apps OR application*)) OR AB((mobile OR smartphone* OR "cell phone*" OR cellphone*) N3 (app OR apps OR application*))
8. TI("mobile health" OR "m-health" OR mhealth OR "m health") OR AB("mobile health" OR "m-health" OR mhealth OR "m health")
9. S3 OR S6 OR S7 OR S8
10. TI((app OR apps) N2 (develop* OR lifecycle OR design*)) OR AB((app OR apps) N2 (develop* OR lifecycle OR design*))
11. TI((content OR functionalit*) N2 (contribut* OR creat* OR develop*)) OR AB((content OR functionalit*) N2 (contribut* OR creat* OR develop*))
12. TI(concept* N2 (develop* OR generat*)) OR AB(concept* N2 (develop* OR generat*))
13. TI(design N2 (thinking OR process* OR criteria)) OR AB(design N2 (thinking OR process* OR criteria))
14. TI(ideate OR ideates OR ideation OR infrastructure OR planning OR prototyp* OR testing) OR AB(ideate OR ideates OR ideation OR infrastructure OR planning OR prototyp* OR testing)
15. TI(idea* N2 (screening OR generat* OR analy*)) OR AB(idea* N2 (screening OR generat* OR analy*))
16. TI(interface N2 (design* OR develop* OR creat*)) OR AB(interface N2 (design* OR develop* OR creat*))
17. TI(project* N2 plan*) OR AB(project* N2 plan*)
18. TI(product* N2 (develop* OR lifecycle OR design*)) OR AB(product* N2 (develop* OR lifecycle OR design*))
19. TI((research N1 development) OR "r&d" OR "r and d") OR AB((research N1 development) OR "r&d" OR "r and d")
20. TI("subject matter expert*" OR SME OR SMEs) OR AB("subject matter expert*" OR SME OR SMEs)
21. TI((alpha OR beta OR marketability OR concept*) N1 test*) OR AB((alpha OR beta OR marketability OR concept*) N1 test*)
22. S10 OR S11 OR S12 OR S13 OR S14 OR S15 OR S16 OR S17 OR S18 OR S19 OR S20 OR S21
23. (MH "Nurses")
24. (MH "Nursing Staff, Hospital")
25. (MH "Students, Nursing+")
26. TI(nurse OR nurses OR nursing OR RN OR RNs OR APN OR APNs OR NP OR NPs) OR AB(nurse OR nurses OR nursing OR RN OR RNs OR APN OR APNs OR NP OR NPs)
27. S23 OR S24 OR S25 OR S26
28. S9 AND S22 AND S27

Cochrane via Wiley

1. MeSH descriptor: [Telemedicine] explode all trees
2. (mobile or app or apps or application*):ti,ab,kw
3. #1 AND #2
4. MeSH descriptor: [Smartphone] explode all trees
5. (app or apps or application*):ti,ab,kw
6. 4 and 5
7. ((mobile or smartphone* or "cell phone*" or cellphone*) NEAR/3 (app or apps or application*)):ti,ab,kw
8. ("mobile health" or "m-health" or mhealth or "m health"):ti,ab,kw
9. #3 OR #6 OR #7 OR #8
10. ((app OR apps) NEAR/2 (develop* OR lifecycle OR design*)):ti,ab,kw
11. ((content OR functionalit*) NEAR/2 (contribut* OR creat* OR develop*)):ti,ab,kw
12. (concept* NEAR/2 (develop* OR generat*)):ti,ab,kw
13. (design NEAR/2 (thinking OR process* OR criteria)):ti,ab,kw
14. (ideate OR ideates OR ideation OR infrastructure OR planning OR prototyp* OR testing):ti,ab,kw
15. (idea* NEAR/2 (screening OR generat* OR analy*)):ti,ab,kw
16. (interface NEAR/2 (design* OR develop* OR creat*)):ti,ab,kw
17. (project* NEAR/2 plan*):ti,ab,kw
18. (product* NEAR/2 (develop* OR lifecycle OR design*)):ti,ab,kw
19. ((research NEAR/1 development) OR "r&d" OR "r and d"):ti,ab,kw
20. ("subject matter expert*" OR SME OR SMEs):ti,ab,kw
21. ((alpha OR beta OR marketability OR concept*) NEAR/1 test*):ti,ab,kw
22. {OR #10-#21}
23. MeSH descriptor: [Nurses] explode all trees
24. MeSH descriptor: [Nursing Staff] explode all trees
25. MeSH descriptor: [Students, Nursing] explode all trees
26. MeSH descriptor: [Nursing] explode all trees
27. (nurse or nurses or nursing or RN or RNs or APN or APNs or NP or NPs):ti,ab,kw
28. {OR #23-#27}
29. #9 AND #22 AND #28
30. MeSH descriptor: [Animals] explode all trees
31. MeSH descriptor: [Humans] explode all trees
32. 30 NOT 31
33. 29 NOT 32

Compendex via Engineering Village

((((({Telemedicine} WN CV) OR ({Health care} WN CV))) AND (((mobile or app or apps or application*)) WN KY)) OR ((((({Smartphones} WN CV)))) AND (((app or apps or application*)) WN KY)) OR ((mobile NEAR/3 app) WN KY) OR ((mobile NEAR/3 apps) WN KY) OR ((mobile NEAR/3 application*) WN KY) OR ((smartphone* NEAR/3 app) WN KY) OR ((smartphone* NEAR/3 apps) WN KY) OR ((smartphone* NEAR/3 application*) WN KY) OR (("cell phone*" NEAR/3 app) WN KY) OR (("cell phone*" NEAR/3 apps) WN KY) OR (("cell phone*" NEAR/3 application*) WN KY) OR ((cellphone* NEAR/3 app) WN KY) OR ((cellphone* NEAR/3 apps) WN KY) OR ((cellphone* NEAR/3 application*) WN KY) OR (("mobile health" or "m-health" or mhealth or "m health") WN KY) OR (((({mHealth} WN CV))))) AND ((({Design} WN CV)) OR ((app NEAR/2 develop*) WN KY) OR ((app NEAR/2 lifecycle) WN KY) OR ((app NEAR/2 design*) WN KY) OR ((apps NEAR/2 develop*) WN KY) OR ((apps NEAR/2 lifecycle) WN KY) OR ((apps NEAR/2 design*) WN KY) OR ((content NEAR/2 contribut*) WN KY) OR ((content NEAR/2 creat*) WN KY) OR ((content

NEAR/2 develop*) WN KY) OR ((functionalit* NEAR/2 contribut*) WN KY) OR ((functionalit* NEAR/2 creat*) WN KY) OR ((functionalit* NEAR/2 develop*) WN KY) OR ((concept* NEAR/2 develop*) WN KY) OR ((concept* NEAR/2 generat*) WN KY) OR ((design NEAR/2 thinking) WN KY) OR ((design NEAR/2 process*) WN KY) OR ((design NEAR/2 criteria) WN KY) OR ((idea* NEAR/2 screening) WN KY) OR ((idea* NEAR/2 generat*) WN KY) OR ((idea* NEAR/2 analy*) WN KY) OR ((interface NEAR/2 design*) WN KY) OR ((interface NEAR/2 develop*) WN KY) OR ((interface NEAR/2 creat*) WN KY) OR ((project* NEAR/2 plan*) WN KY) OR ((product* NEAR/2 develop*) WN KY) OR ((product* NEAR/2 lifecycle) WN KY) OR ((product* NEAR/2 design*) WN KY) OR ((research NEAR/1 development) WN KY) OR ((alpha NEAR/1 test*) WN KY) OR ((beta NEAR/1 test*) WN KY) OR ((marketability NEAR/1 test*) WN KY) OR ((concept* NEAR/1 test*) WN KY) OR (("r&d" OR "r and d" OR "subject matter expert*" OR SME OR SMEs OR ideate OR ideates OR ideation OR infrastructure OR planning OR prototyp* OR testing) WN KY) OR (({Product development} WN CV) OR ({Software prototyping} WN CV) OR ({Research and development management} WN CV))) AND ((nurse or nurses or nursing or RN or RNs or APN or APNs or NP or NPs) WN KY)

Education Source via EBSCO

1. TI((mobile OR smartphone* OR "cell phone*" OR cellphone*) N3 (app OR apps OR application*)) OR AB((mobile OR smartphone* OR "cell phone*" OR cellphone*) N3 (app OR apps OR application*)) OR KW((mobile OR smartphone* OR "cell phone*" OR cellphone*) N3 (app OR apps OR application*))
2. TI("mobile health" OR "m-health" OR mhealth OR "m health") OR AB("mobile health" OR "m-health" OR mhealth OR "m health") OR KW("mobile health" OR "m-health" OR mhealth OR "m health")
3. S1 OR S2
4. TI((app OR apps) N2 (develop* OR lifecycle OR design*)) OR AB((app OR apps) N2 (develop* OR lifecycle OR design*)) OR KW((app OR apps) N2 (develop* OR lifecycle OR design*))
5. TI((content OR functionalit*) N2 (contribut* OR creat* OR develop*)) OR AB((content OR functionalit*) N2 (contribut* OR creat* OR develop*)) OR KW((content OR functionalit*) N2 (contribut* OR creat* OR develop*))
6. TI(concept* N2 (develop* OR generat*)) OR AB(concept* N2 (develop* OR generat*)) OR KW(concept* N2 (develop* OR generat*))
7. TI(design N2 (thinking OR process* OR criteria)) OR AB(design N2 (thinking OR process* OR criteria)) OR KW(design N2 (thinking OR process* OR criteria))
8. TI(ideate OR ideates OR ideation OR infrastructure OR planning OR prototyp* OR testing) OR AB(ideate OR ideates OR ideation OR infrastructure OR planning OR prototyp* OR testing) OR KW(ideate OR ideates OR ideation OR infrastructure OR planning OR prototyp* OR testing)
9. TI(idea* N2 (screening OR generat* OR analy*)) OR AB(idea* N2 (screening OR generat* OR analy*)) OR KW(idea* N2 (screening OR generat* OR analy*))
10. TI(interface N2 (design* OR develop* OR creat*)) OR AB(interface N2 (design* OR develop* OR creat*)) OR KW(interface N2 (design* OR develop* OR creat*))
11. TI(project* N2 plan*) OR AB(project* N2 plan*) OR KW(project* N2 plan*)
12. TI(product* N2 (develop* OR lifecycle OR design*)) OR AB(product* N2 (develop* OR lifecycle OR design*)) OR KW(product* N2 (develop* OR lifecycle OR design*))
13. TI((research N1 development) OR "r&d" OR "r and d") OR AB((research N1 development) OR "r&d" OR "r and d") OR KW((research N1 development) OR "r&d" OR "r and d")
14. TI("subject matter expert*" OR SME OR SMEs) OR AB("subject matter expert*" OR SME OR SMEs) OR KW("subject matter expert*" OR SME OR SMEs)

15. TI((alpha OR beta OR marketability OR concept*) N1 test*) OR AB((alpha OR beta OR marketability OR concept*) N1 test*) OR KW((alpha OR beta OR marketability OR concept*) N1 test*)
16. DE "Computer software development"
17. S4 OR S5 OR S6 OR S7 OR S8 OR S9 OR S10 OR S11 OR S12 OR S13 OR S14 OR S15 OR S16
18. DE "Nursing students"
19. DE "Nurse educators"
20. DE "Nursing teachers"
21. TI(nurse OR nurses OR nursing OR RN OR RNs OR APN OR APNs OR NP OR NPs) OR AB(nurse OR nurses OR nursing OR RN OR RNs OR APN OR APNs OR NP OR NPs) OR KW(nurse OR nurses OR nursing OR RN OR RNs OR APN OR APNs OR NP OR NPs)
22. S18 OR S19 OR S20 OR S21
23. S3 AND S17 AND S22

Embase via Ovid

1. exp telemedicine/
2. (mobile or app or apps or application*).tw,kw
3. 1 and 2
4. exp mobile phone/
5. (app or apps or application*).tw,kw
6. 4 and 5
7. ((mobile or smartphone* or "cell phone*" or cellphone*) adj3 (app or apps or application*)).tw,kw
8. ("mobile health" or "m-health" or mhealth or "m health").tw,kw
9. OR/3,6-8
10. ((app OR apps) adj2 (develop* OR lifecycle OR design*)).tw,kw
11. ((content OR functionalit*) adj2 (contribut* OR creat* OR develop*)).tw,kw
12. (concept* adj2 (develop* OR generat*)).tw,kw
13. (design adj2 (thinking OR process* OR criteria)).tw,kw
14. (ideate OR ideates OR ideation OR infrastructure OR planning OR prototyp* OR testing).tw,kw
15. (idea* adj2 (screening OR generat* OR analy*)).tw,kw
16. (interface adj2 (design* OR develop* OR creat*)).tw,kw
17. (project* adj2 plan*).tw,kw
18. (product* adj2 (develop* OR lifecycle OR design*)).tw,kw
19. ((research adj1 development) OR "r&d" OR "r and d").tw,kw
20. ("subject matter expert*" OR SME OR SMEs).tw,kw
21. ((alpha OR beta OR marketability OR concept*) adj1 test*).tw,kw
22. OR/10-21
23. exp nurse/
24. exp nursing staff/
25. exp nursing student/
26. exp nursing/
27. (nurse or nurses or nursing or RN or RNs or APN or APNs or NP or NPs).tw,kw
28. OR/23-27
29. 9 AND 22 AND 28
30. exp animal/
31. exp human/
32. 30 NOT 31
33. 29 NOT 32

34. ..dedup 33

ERIC via EBSCO

1. DE "Handheld Devices"
2. TI(app OR apps OR application*) OR AB(app OR apps OR application*) OR KW(app OR apps OR application*)
3. S1 AND S2
4. TI((mobile OR smartphone* OR "cell phone*" OR cellphone*) N3 (app OR apps OR application*)) OR AB((mobile OR smartphone* OR "cell phone*" OR cellphone*) N3 (app OR apps OR application*)) OR KW((mobile OR smartphone* OR "cell phone*" OR cellphone*) N3 (app OR apps OR application*))
5. TI("mobile health" OR "m-health" OR mhealth OR "m health") OR AB("mobile health" OR "m-health" OR mhealth OR "m health") OR KW("mobile health" OR "m-health" OR mhealth OR "m health")
6. S3 OR S4 OR S5
7. TI((app OR apps) N2 (develop* OR lifecycle OR design*)) OR AB((app OR apps) N2 (develop* OR lifecycle OR design*)) OR KW((app OR apps) N2 (develop* OR lifecycle OR design*))
8. TI((content OR functionalit*) N2 (contribut* OR creat* OR develop*)) OR AB((content OR functionalit*) N2 (contribut* OR creat* OR develop*)) OR KW((content OR functionalit*) N2 (contribut* OR creat* OR develop*))
9. TI(concept* N2 (develop* OR generat*)) OR AB(concept* N2 (develop* OR generat*)) OR KW(concept* N2 (develop* OR generat*))
10. TI(design N2 (thinking OR process* OR criteria)) OR AB(design N2 (thinking OR process* OR criteria)) OR KW(design N2 (thinking OR process* OR criteria))
11. TI(ideate OR ideates OR ideation OR infrastructure OR planning OR prototyp* OR testing) OR AB(ideate OR ideates OR ideation OR infrastructure OR planning OR prototyp* OR testing) OR KW(ideate OR ideates OR ideation OR infrastructure OR planning OR prototyp* OR testing)
12. TI(idea* N2 (screening OR generat* OR analy*)) OR AB(idea* N2 (screening OR generat* OR analy*)) OR KW(idea* N2 (screening OR generat* OR analy*))
13. TI(interface N2 (design* OR develop* OR creat*)) OR AB(interface N2 (design* OR develop* OR creat*)) OR KW(interface N2 (design* OR develop* OR creat*))
14. TI(project* N2 plan*) OR AB(project* N2 plan*) OR KW(project* N2 plan*)
15. TI(product* N2 (develop* OR lifecycle OR design*)) OR AB(product* N2 (develop* OR lifecycle OR design*)) OR KW(product* N2 (develop* OR lifecycle OR design*))
16. TI((research N1 development) OR "r&d" OR "r and d") OR AB((research N1 development) OR "r&d" OR "r and d") OR KW((research N1 development) OR "r&d" OR "r and d")
17. TI("subject matter expert*" OR SME OR SMEs) OR AB("subject matter expert*" OR SME OR SMEs) OR KW("subject matter expert*" OR SME OR SMEs)
18. TI((alpha OR beta OR marketability OR concept*) N1 test*) OR AB((alpha OR beta OR marketability OR concept*) N1 test*) OR KW((alpha OR beta OR marketability OR concept*) N1 test*)
19. DE "Computer System Design" OR DE "Client Server Architecture"
20. S7 OR S8 OR S9 OR S10 OR S11 OR S12 OR S13 OR S14 OR S15 OR S16 OR S17 OR S18 OR S19
21. DE "Nurses" OR DE "School Nurses"
22. DE "Nursing Students"
23. DE "Nursing"
24. DE "Nursing Education" OR DE "Nursing Research"

25. TI(nurse OR nurses OR nursing OR RN OR RNs OR APN OR APNs OR NP OR NPs) OR AB(nurse OR nurses OR nursing OR RN OR RNs OR APN OR APNs OR NP OR NPs) OR KW(nurse OR nurses OR nursing OR RN OR RNs OR APN OR APNs OR NP OR NPs)
26. S21 OR S22 OR S23 OR S24 OR S25
27. S6 AND S20 AND S26

Global Index Medicus

((tw:((mobile OR smartphone* OR "cell phone*" OR cellphone*) AND (app OR apps OR application*))) OR (tw:("mobile health" OR "m-health" OR mhealth OR "m health")) OR ((mh:("Telemedicine")) AND (tw:(mobile OR app OR apps OR application*))) OR ((mh:("Smartphone")) AND (tw:(app OR apps OR application*)))) AND ((tw:((app OR apps) AND (develop* OR lifecycle OR design*))) OR (tw:((content OR functionalit*) AND (contribut* OR creat* OR develop*))) OR (tw:(concept* AND (develop* OR generat*))) OR (tw:(design AND (thinking OR process* OR criteria))) OR (tw:(ideate OR ideates OR ideation OR infrastructure OR planning OR prototyp* OR testing)) OR (tw:(idea* AND (screening OR generat* OR analy*))) OR (tw:(interface AND (design* OR develop* OR creat*))) OR (tw:(project* AND plan*)) OR (tw:(product* AND (develop* OR lifecycle OR design*))) OR (tw:((research AND development) OR "r&d" OR "r and d")) OR (tw:("subject matter expert*" OR SME OR SMEs)) OR (tw:((alpha OR beta OR marketability OR concept*) AND test*))) AND ((mh:("Nurses")) OR (mh:("Nursing Staff")) OR (mh:("Students, Nursing")) OR (mh:("Nursing")) OR (tw:(nurse OR nurses OR nursing OR RN OR RNs OR APN OR APNs OR NP OR NPs)))

Google Scholar

allintitle: (nurses OR nurse OR nursing OR RNs OR APNs OR NPs)("mobile health" OR "mobile applications" OR apps OR app OR devices OR mhealth OR "m-health" OR "m health")(designing OR design OR development OR developing OR ideation OR prototyping OR testing OR functionality OR "subject matter experts" OR SMEs OR research)

IEEE Xplore

("Mesh_Terms":Nurses OR "Mesh_Terms":Nursing Staff OR "Mesh_Terms":Students, Nursing OR "Mesh_Terms":Nursing OR ("All Metadata":nurse) OR ("All Metadata":nurses) OR ("All Metadata":nursing) OR ("All Metadata":RN) OR ("All Metadata":RNs) OR ("All Metadata":APN) OR ("All Metadata":APNs) OR ("All Metadata":NP) OR ("All Metadata":NPs)) AND (("All Metadata": mobile NEAR/3 app) OR ("All Metadata": mobile NEAR/3 apps) OR ("All Metadata": mobile NEAR/3 application*) OR ("All Metadata": smartphone NEAR/3 app) OR ("All Metadata": smartphone NEAR/3 apps) OR ("All Metadata": smartphone NEAR/3 application*) OR ("All Metadata": "cell phone" NEAR/3 app) OR ("All Metadata": "cell phone" NEAR/3 apps) OR ("All Metadata": "cell phone" NEAR/3 application*) OR ("All Metadata": cellphone NEAR/3 app) OR ("All Metadata": cellphone NEAR/3 apps) OR ("All Metadata": cellphone NEAR/3 application*) OR ((("IEEE Terms":telemedicine OR "Publication Topics":telemedicine OR "Mesh_Terms":telemedicine) AND (("All Metadata":mobile) OR ("All Metadata":app) OR ("All Metadata":apps) OR ("All Metadata":application*))) OR (("IEEE Terms":smart phones OR "Mesh_Terms":Smartphone) AND (("All Metadata":app) OR ("All Metadata":apps) OR ("All Metadata":application*))))) AND (("All Metadata":"design thinking") OR ("All Metadata":lifecycle) OR ("All Metadata":development) OR ("All Metadata":"design process") OR ("All Metadata":"design processes") OR ("All Metadata":"design criteria") OR ("All Metadata":ideate) OR ("All Metadata":ideation) OR ("All Metadata":ideates) OR ("All Metadata":infrastructure) OR

("All Metadata":planning) OR ("All Metadata":prototype) OR ("All Metadata":prototyping) OR ("All Metadata":testing) OR ("All Metadata":research) OR ("All Metadata":"r&d") OR ("All Metadata":"r and d") OR ("All Metadata":"subject matter expert") OR ("All Metadata":"subject matter experts") OR ("All Metadata":sme) OR ("All Metadata":smes))

Medline via Ovid

1. exp Telemedicine/
2. (mobile or app or apps or application*).tw,kw
3. 1 and 2
4. exp Smartphone
5. (app or apps or application*).tw,kw
6. 4 and 5
7. ((mobile or smartphone* or "cell phone*" or cellphone*) adj3 (app or apps or application*)).tw,kw
8. ("mobile health" or "m-health" or mhealth or "m health").tw,kw
9. OR/3,6-8
10. ((app OR apps) adj2 (develop* OR lifecycle OR design*)).tw,kw
11. ((content OR functionalit*) adj2 (contribut* OR creat* OR develop*)).tw,kw
12. (concept* adj2 (develop* OR generat*)).tw,kw
13. (design adj2 (thinking OR process* OR criteria)).tw,kw
14. (ideate OR ideates OR ideation OR infrastructure OR planning OR prototyp* OR testing).tw,kw
15. (idea* adj2 (screening OR generat* OR analy*)).tw,kw
16. (interface adj2 (design* OR develop* OR creat*)).tw,kw
17. (project* adj2 plan*).tw,kw
18. (product* adj2 (develop* OR lifecycle OR design*)).tw,kw
19. ((research adj1 development) OR "r&d" OR "r and d").tw,kw
20. ("subject matter expert*" OR SME OR SMEs).tw,kw
21. ((alpha OR beta OR marketability OR concept*) adj1 test*).tw,kw
22. OR/10-21
23. exp Nurses
24. exp Nursing Staff
25. exp Students, Nursing
26. exp Nursing
27. (nurse or nurses or nursing or RN or RNs or APN or APNs or NP or NPs).tw,kw
28. OR/23-27
29. 9 AND 22 AND 28
30. exp Animals/
31. exp Humans/
32. 30 NOT 31
33. 29 NOT 32
34. ..dedup 33

PsycINFO via Ovid

1. exp telemedicine/
2. (mobile or app or apps or application*).tw
3. 1 and 2
4. exp mobile devices/

5. (app or apps or application*).tw
6. 4 and 5
7. ((mobile or smartphone* or "cell phone*" or cellphone*) adj3 (app or apps or application*)).tw
8. ("mobile health" or "m-health" or mhealth or "m health").tw
9. OR/3,6-8
10. ((app OR apps) adj2 (develop* OR lifecycle OR design*)).tw
11. ((content OR functionalit*) adj2 (contribut* OR creat* OR develop*)).tw
12. (concept* adj2 (develop* OR generat*)).tw
13. (design adj2 (thinking OR process* OR criteria)).tw
14. (ideate OR ideates OR ideation OR infrastructure OR planning OR prototyp* OR testing).tw
15. exp human machine systems design/
16. (idea* adj2 (screening OR generat* OR analy*)).tw
17. (interface adj2 (design* OR develop* OR creat*)).tw
18. (project* adj2 plan*).tw
19. (product* adj2 (develop* OR lifecycle OR design*)).tw
20. ((research adj1 development) OR "r&d" OR "r and d").tw
21. ("subject matter expert*" OR SME OR SMEs).tw
22. ((alpha OR beta OR marketability OR concept*) adj1 test*).tw
23. OR/10-22
24. exp nurses/
25. exp nursing/
26. (nurse or nurses or nursing or RN or RNs or APN or APNs or NP or NPs).tw
27. OR/24-26
28. 9 AND 23 AND 27
29. ..dedup 28

PubMed

(("Telemedicine"[Mesh] AND (mobile[tw] OR app[tw] OR apps[tw] OR application*[tw])) OR ("Smartphone"[Mesh] AND (app[tw] OR apps[tw] OR application*[tw])) OR ((mobile[tw] OR smartphone*[tw] OR "cell phone*"[tw] OR cellphone*) AND (app[tw] OR apps[tw] OR application*[tw])) OR "mobile health"[tw] OR "m-health"[tw] OR mhealth[tw] OR "m health"[tw]) AND (((app[tw] OR apps[tw]) AND (develop*[tw] OR lifecycle[tw] OR design*[tw])) OR ((content[tw] OR functionalit*) AND (contribut*[tw] OR creat*[tw] OR develop[tw])) OR (concept* AND (develop*[tw] OR generat*[tw])) OR (design AND (thinking[tw] OR process*[tw] OR criteria[tw])) OR ideate[tw] OR ideates[tw] OR ideation[tw] OR infrastructure[tw] OR planning[tw] OR prototyp*[tw] OR testing[tw] OR (idea* AND (screening[tw] OR generat*[tw] OR analy*[tw])) OR (interface AND (design*[tw] OR develop*[tw] OR creat*[tw])) OR (project*[tw] AND plan*[tw]) OR (product*[tw] AND (develop*[tw] OR lifecycle[tw] OR design*[tw])) OR ((research[tw] AND development[tw]) OR "r&d"[tw] OR "r and d") OR "subject matter expert*"[tw] OR SME[tw] OR SMEs[tw] OR ((alpha[tw] OR beta[tw] OR marketability[tw] OR concept*[tw]) AND test*[tw])) AND ("Nurses"[Mesh] OR "Nursing Staff"[Mesh] OR "Students, Nursing"[Mesh] OR "Nursing"[Mesh] OR nurse[tw] OR nurses[tw] OR nursing[tw] OR RN[tw] OR RNs[tw] OR APN[tw] OR APNs[tw] OR NP[tw] OR NPs[tw]) NOT ("Animals"[Mesh] NOT "Humans"[Mesh])

Scopus

((INDEXTERMS ("Telemedicine") AND TITLE-ABS-KEY (mobile OR app OR apps OR application*)) OR ((INDEXTERMS ("Smartphone") OR INDEXTERMS ("Mobile Phone")) AND TITLE-ABS-KEY (app

OR apps OR application*)) OR TITLE-ABS-KEY ((mobile OR smartphone* OR "cell phone*" OR cellphone*) W/3 (app OR apps OR application*)) OR TITLE-ABS-KEY ("mobile health" OR "m-health" OR mhealth OR "m health")) AND TITLE-ABS-KEY (((app OR apps) W/2 (develop* OR lifecycle OR design*)) OR ((content OR functionalit*) W/2 (contribut* OR creat* OR develop*)) OR (concept* W/2 (develop* OR generat*)) OR (design W/2 (thinking OR process* OR criteria)) OR (ideate OR ideates OR ideation OR infrastructure OR planning OR prototyp* OR testing) OR (idea* W/2 (screening OR generat* OR analy*)) OR (interface W/2 (design* OR develop* OR creat*)) OR (project* W/2 plan*) OR (product* W/2 (develop* OR lifecycle OR design*)) OR ((research W/1 development) OR "r&d" OR "r and d") OR ("subject matter expert*" OR sme OR smes) OR ((alpha OR beta OR marketability OR concept*) W/1 test*)) AND (INDEXTERMS ("Nurses") OR INDEXTERMS ("Nursing Staff") OR INDEXTERMS ("Students, Nursing") OR INDEXTERMS ("Nursing Student") OR INDEXTERMS ("Nursing") OR TITLE-ABS-KEY (nurse OR nurses OR nursing OR rn OR rns OR apn OR apns OR np OR nps)) AND NOT (INDEXTERMS ("Animals") AND NOT INDEXTERMS ("Humans"))

Web of Science

1. TS=((mobile or smartphone* or "cell phone*" or cellphone*) NEAR/3 (app or apps or application*))
2. TS=("mobile health" or "m-health" or mhealth or "m health")
3. #1 OR #2
4. TS=((app OR apps) NEAR/2 (develop* OR lifecycle OR design*))
5. TS=((content OR functionalit*) NEAR/2 (contribut* OR creat* OR develop*))
6. TS=(concept* NEAR/2 (develop* OR generat*))
7. TS=(design NEAR/2 (thinking OR process* OR criteria))
8. TS=(ideate OR ideates OR ideation OR infrastructure OR planning OR prototyp* OR testing)
9. TS=(idea* NEAR/2 (screening OR generat* OR analy*))
10. TS=(interface NEAR/2 (design* OR develop* OR creat*))
11. TS=(project* NEAR/2 plan*)
12. TS=(product* NEAR/2 (develop* OR lifecycle OR design*))
13. TS=((research NEAR/1 development) OR "r&d" OR "r and d")
14. TS=("subject matter expert*" OR SME OR SMEs)
15. TS=((alpha OR beta OR marketability OR concept*) NEAR/1 test*)
16. #4 OR #5 OR #6 OR #7 OR #8 OR #9 OR #10 OR #11 OR #12 OR #13 OR #14 OR #15
17. TS=(nurse or nurses or nursing or RN or RNs or APN or APNs or NP or NPs)
18. #3 AND #16 AND #17
